# Acceptance of Self-Sampling by Women Not Regularly Participating in Cervical Cancer Screening in Areas with Low Medical Density: A Qualitative Study within the French CapU4 Trial

**DOI:** 10.3390/cancers16112066

**Published:** 2024-05-30

**Authors:** Johane Le Goff, Anne-Sophie Le Duc-Banaszuk, Caroline Lefeuvre, Adeline Pivert, Alexandra Ducancelle, Hélène De Pauw, Marc Arbyn, Aubeline Vinay, Franck Rexand-Galais

**Affiliations:** 1 University of Angers, CLiPsy, SFR CONFLUENCES, F-49000 Angers, France; aubeline.vinay@univ-angers.fr (A.V.); franck.rexand-galais@univ-angers.fr (F.R.-G.); 2 Pays de la Loire Regional Cancer Screening Coordination Center (CRCDC Pays de La Loire), F-49000 Angers, France; as.banaszuk@depistagecancers.fr; 3 University of Angers, CHU Angers, HIFIH, SFR ICAT, F-49000 Angers, France; caroline.lefeuvre@chu-angers.fr (C.L.); adeline.pivert.chu@gmail.com (A.P.); alducancelle@chu-angers.fr (A.D.); 4 Unit Cancer Epidemiology, Belgian Cancer Center, Sciensano, B1050 Brussels, Belgium; helene.depauw@sciensano.be (H.D.P.); marc.arbyn@sciensano.be (M.A.); 5 Department of Human Structure and Repair, Faculty of Medicine and Health Sciences, Ghent University, B9000 Ghent, Belgium

**Keywords:** cervical cancer, screening strategies, under-screened women, low medical density, urinary self-sampling, vaginal self-sampling, qualitative study

## Abstract

**Simple Summary:**

In spite of effective preventive tools, an estimated 3159 women were still diagnosed with cervical cancer (CC) in France in 2023, and 1117 died from the disease. We investigated women’s opinions on CC screening and how the offer of self-sampling kits could address barriers impeding the outreach of under-screened populations in three rural French administrative departments with low medical density and/or low screening participation rates. The qualitative study confirmed acceptability to receive self-sampling kits but also highlighted the need for clear, adapted, and simple instructions and information (preferably from a healthcare professional) on what to do in case of a positive result. Women embarrassed by genital self-examination may prefer urine collection kits.

**Abstract:**

Cervical cancer (CC) was diagnosed in 3159 women in France in 2023, and 1117 died from it. Organized screening for cervical cancer is potentially very effective for participating women. However, reaching under-screened populations remains a major challenge. The present qualitative study explored women’s opinions on what discourages or encourages them to participate in CC screening and assessed the acceptability of two experimental strategies (urinary or vaginal self-sampling kits) to increase the screening coverage in three rural French administrative departments with low medical density and/or low screening participation rates. Forty-eight semi-structured interviews and four focus groups were conducted by a team of psychologists. Results showed that the participants accepted at-home self-sampling to reach non-participating women in medically underserved areas. However, they suggested that the type of kit sent should be adapted to the patient’s profile (embarrassment from earlier exams, cultural aspects, fear of invasiveness, etc.), and that kits should be simple to use (in understandable language taking sociocultural aspects into account). Women wished to be assured that testing on self-samples is accurate and needed information about further actions in case of a positive result.

## 1. Introduction

Cervical cancer (CC) is the fourth most frequently diagnosed cancer in women worldwide [1], and yet it is a preventable malignancy, primarily through the vaccination of adolescents and secondarily through HPV screening every 5–10 years starting at the age of 30 years [2]. Screening based on HPV detection offers 60–70% greater protection than cytology for invasive cancers [3]. Due to the international importance of the phenomenon (604,000 new cases and 342,000 deaths in 2020), the World Health Organization has launched a global initiative to accelerate the elimination of CC, building it upon three important pillars: vaccination, screening, and treatment [4]. However, substantial geographical and socioeconomic inequalities persist worldwide in relation to screening [5], as do major inequalities in incidence, mortality, and access to quality care between European countries [6]. The regular use of cancer screening therefore remains insufficient.

In France, around 3000 new cases are diagnosed every year. In 2018, despite the implementation of various prevention policies over the preceding two decades to encourage regular use of cancer screening [7], CC was responsible for 1117 deaths [8]. In France, organized screening has been introduced to reach under-screened populations. It includes invitations to have cervical tests for women who have not been screened or who have missed the screening deadline. Since 2019, the French National Authority for Health (*Haute Autorité de Santé*—HAS) has recommended HPV testing every 5 years after an initial negative HPV test. Also, vaginal self-sampling (VSS) may be an alternative to clinician sampling; it should be offered to women who have not been screened or who have been inadequately screened from the age of 30 [9]. Self-sampling has been shown to be as reliable as clinician sampling [10]. Meta-analyses have demonstrated that validated PCR-based HPV tests on self-samples have similar sensitivity and slightly lower specificity compared to clinician-collected samples to detect cervical intraepithelial neoplasia grade 2 (CIN 2) or CIN grade 3 [10]. The clinical accuracy of HPV testing on VSS was investigated in the VALHUDES trial (VALidation of HUman papillomavirus assays and collection DEvices for Self-samples and urine samples), and one of the most often used and commercially available brushes for VSS is the FLOQSwabs^®^ vaginal self-sampling device (Copan Diagnostics, Brescia, Italy), a dry swab [11,12]. Furthermore, VSS represents an opportunity to reach under-screened women [13,14] and, moreover still, is better accepted by women, who generally prefer it to cervical-uterine sampling carried out by healthcare professionals [15,16]. Strangely though, only eight countries worldwide recommend VSS as a means of reaching under-screened populations [17]. Additionally, studies have demonstrated the effectiveness of urinary self-sampling (USS) in increasing women’s adherence to CC screening. This method, too, has been shown to be effective in detecting precancerous and cancerous lesions of the cervix [18]. The VALHUDES study has clinically evaluated and validated the use of HPV assays on USS collected with the Colli-Pee device [19] and has shown good performance of HPV assays on USS versus on clinician-collected samples [20,21]. Colli-Pee is a device that collects only the first-void fraction and mixes the collected urine with a conserving liquid. However, USS is currently only proposed in research protocols [20,22] and not as a screening proposition, even though it also could provide an alternative to cervical samples and possibly extend screening coverage to non-participating women in medically underserved areas.

Currently, there is little evidence concerning the effectiveness of sending USS kits, compared to VSS kits or conventional invitations, noting that low response rates have been observed among women who receive the latter [23]. In France’s Pays de la Loire administrative region, CC screening coverage was 62.4% in 2018–2020 [24], despite the fact that screening can be done not only by gynecologists but also by general practitioners and midwives. There are disparities between the region’s administrative departments, with the mainly rural Mayenne and Sarthe departments having the lowest rates, respectively, 60.7% and 58.2% [25]. Additionally, the Vendée department is showing declining medical density, which may affect CC screening coverage: 64.2% [26].

The aim of the work presented here was to gather women’s opinions on CC screening and on experimental self-sampling strategies to reach under-screened populations in these three rural French departments with low medical density and/or low screening participation rates.

## 2. Materials and Methods

The aim of the randomized CapU4 study, whose methodology has previously been presented [27], is to evaluate the effectiveness of two experimental invitation strategies (offering USS or VSS kits) in reaching under-screened populations and to compare them with the current invitation strategy (control arm) in French rural administrative departments with low medical density and/or low CC screening participation. In the control arm, women receive a conventional invitation letter sent by post to the home address of eligible women recommending them to make an appointment to a doctor or a midwife for the collection of a cervical specimen. The two experimental interventions correspond to: (1) eligible women who received a free VSS kit (FLOQSwabs^®^, Copan Diagnostics, Brescia, Italy) at their home address in addition to the conventional invitation letter; and (2) eligible women who received a free urine collection kit (Colli-Pee device, Novosanis, Wijnegem, Belgium) at their home address in addition to the conventional invitation letter. The target population comprises women aged between 30 and 65 years who had not been screened for more than three years and who had not responded to a screening invitation letter in the last 12 months.

The present qualitative work was focused on what discourages or encourages participation in conventional CC screening or USS or VSS screening among women who performed self-sampling tests. Particular attention was paid to the population characterized as under-screened (over 50 years old) and to that considered as vulnerable (economically disadvantaged, receiving complementary care, medically deserted areas, etc.).

This qualitative study was based on the analysis of 48 semi-structured interviews and four focus groups, conducted between April and August 2022 and again between April and August 2023. The CapU4 research project was reviewed and ethically approved by the Sud-Est I Institutional Review Board (2021-123, 25 November 2021, France). The French Data Protection Authority was informed of the use of recordings for the study in September 2021 (ref. 2223607v0). All women participating in the semi-structured interviews and focus groups provided signed, informed consent, and all data were anonymized.

### 2.1. Semi-Structured Interviews and Focus-Group

Semi-structured interviews and focus groups were used to identify the motivations of women who returned self-sampling kits. The women were first questioned on their knowledge of CC, its screening, and the self-screening program. The next step was to establish what they did with the results they received and to evaluate their experience with taking the sample, to measure satisfaction and assess the value placed on the information they received. The interviews and focus groups were designed by psychologists from the CLiPsy Research Unit in the Department of Psychology at the University of Angers (France). They were built on previously explored theoretical elements and practices [28,29,30,31], incorporating specific indicators identified in preceding studies (particularly a lack of time, discomfort with the location, or bad experiences encountered during other types of examinations, beliefs, etc.).

A first session of semi-structured interviews and focus groups was carried out between April and August 2022. The use of focus groups was particularly useful for understanding similarities and differences in participants’ thoughts, views, and emotions [32,33]. This method provided additional information for the second session of semi-structured interviews and focus groups held between April and August 2023. Interviews were conducted until response saturation was reached [34]. The interview guides can be found in the: Appendixes (Appendix A) for the semi-structured interview, p. 12–14; (Appendix B) for the focus group, p. 14–15.

### 2.2. Population

Women aged between 30 and 65 years were surveyed. Demographic and geographic criteria were collected, such as age, level of education, profession, area of residence, presence of a long-term condition (LTC), and whether they benefited from subsidized complementary health insurance (Complémentaire Santé Solidaire—CSS, In France, the “complémentaire santé solidaire” is supplementary health insurance for people of modest means).

### 2.3. Process and Analysis

The self-screening kits, written information on the study and its objectives, and a questionnaire to sound the women’s opinions on CC and the use of the kits were sent as a single mailing by the Pays de la Loire’s regional coordination center for cancer screening (Centre Régional de Coordination des Dépistages des Cancers—CRCDC, a non-profit public health organization offering organized screening for colorectal, breast and cervical cancer). Women wishing to take part in the qualitative study were asked to provide their contact details on the questionnaire and return it to the CRCDC. The CRCDC then provided the psychologists with the contact details of the volunteers. Thereafter, the team of psychologists contacted 241 women by phone for the study, specifically 129 who had received VSS and 112 who had received USS kits.

Interviews and focus groups were analyzed using standard qualitative content analysis [35]. Grounded theory [36] served as a reference for this qualitative approach. The technique of constant comparison between emerging data and previous data was applied. Each participant’s responses were synthesized to reveal the main themes for each age group. This analytical approach made it possible to compare opinions, determine similarities and differences in the data [37,38] and identify specificities according to age group. Two additional interviews per age group were carried out to confirm the results and reach data saturation.

## 3. Results

The sample of semi-structured interviews comprised 48 participants: 24 for VSS, 24 for USS. The interviews lasted between 9 and 27 min (μ = 18.4 min). The four focus groups (FG) were composed of women representative of the initial sample, according to the socio-demographic characteristics defined in the protocol and presented in Table 1 below. The non-agreement of some women in the initial sample to participate led to the recruitment of eight additional participants meeting the protocol requirements. These eight women belonged to the following age groups: two women aged 30–40 years, three women aged 41–50 years, and three women aged 51–65 years. The focus groups lasted between 37 and 56.5 min (μ = 45.6 min). The mean LTC rate in the groups was 12.5%, which corresponded to the mean reported for French women in the age range of the study’s participants [39].

The sociodemographic characteristics of the sample are presented in Table 1.

### 3.1. Barriers to CC Screening

#### 3.1.1. Semi-Structured Interview Data

**Figure 1:**
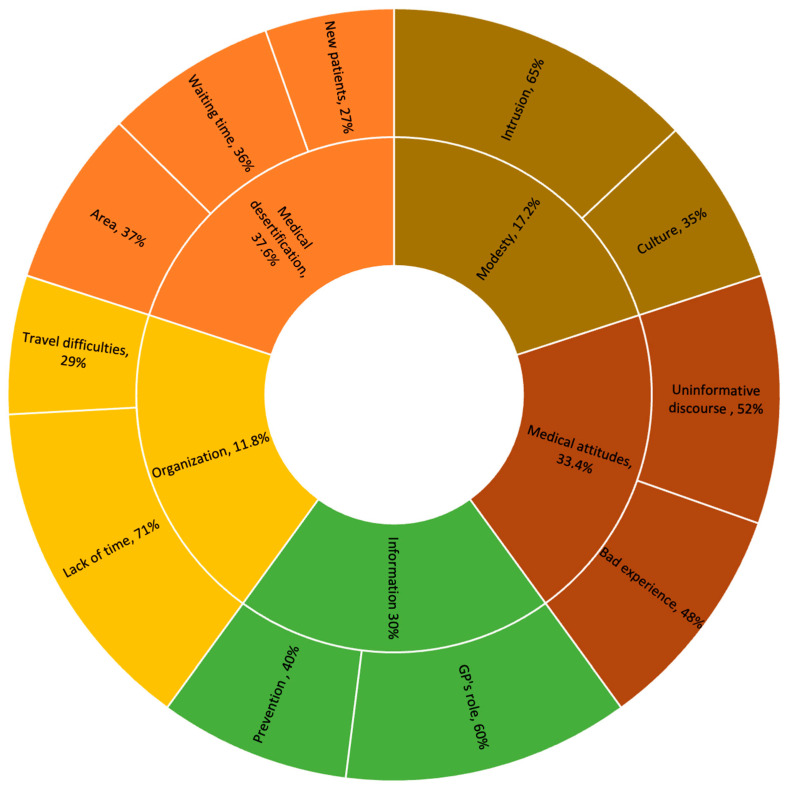
Influencing factors and barriers to cervical screening according to the interviewed women.

All of the interviewed women identified barriers to CC screening. A conventional qualitative content analysis [31] enabled the selection of five general categories furthermore divided into sub-categories to provide a more detailed understanding of the barriers. The importance of the themes raised by women is indicated by percentages (Figure 1).

The three factors with the greatest impact according to the participants were medical desertification (37.6%), medical attitudes (33.4%), and provided information (30%). The least influential factors were modesty (17.2%) and organization (11.8%). Participant citations are used below to illustrate these barriers.

Medical desertification was clearly identified by the participants as the most important barrier to screening (37.6%): “There are so few doctors, it’s a disaster in the countryside” (W14 USS); “I have to drive 50 min to see him, so 50 to go, 50 to come back; I have too much to do for that” (W1 VSS). This desertification also created disproportionately long delays for appointments: “The waiting time is six to nine months. I don’t know what I’ll be doing nine months out. So, I prefer to not go for screening” (W8 VSS). Finally, medical desertification had an influence on the very possibility of receiving regular medical care, as many health professionals were unable to accept new patients: “I haven’t had a GP for six years, they systematically refuse new patients” (W7 VSS); “I don’t get check-ups, there’s no one who’s able to give me check-ups” (W3 USS); “My gynecologist has retired, no one is taking on new patients” (W16 USS).

The second most identified barrier by the participating women was medical attitude attitudes (33.4%), i.e., the behavior and attitudes of healthcare professionals in the context of CC screening. This factor was described as a major obstacle to CC screening, mainly through the uninformative discourse of healthcare professionals: “No one has ever talked to me about CC screening” (W1 USS); “Informing is a big word. My doctor never informed me about anything, we’re the ones who must broach the subject” (W2 USS); “No professional has ever suggested screening to me (W24 VSS). Screening for CC was sometimes mentioned indirectly. For example, one channel for information was care being provided to an adolescent: “I was told about CC through my daughter’s vaccination” (W10 USS). Healthcare professionals’ attitudes were also identified through another prism, that of bad experiences, which can leave a traumatic trace: “I was traumatized by my last smear test, so I didn’t want to do any more” (W13 VSS); “Doctors are neither attentive nor caring” (W6 USS); “There are a whole bunch of doctors who don’t dare say they’re not qualified for smear test” (W20 USS); “The fact that someone else does the procedure for me (...) I consider it rape” (Focus-Group (FG) 1).

Finally, information was the third factor most frequently identified as a hindrance to the screening process (30%). When it did exist, information was perceived as ineffective because not sufficiently consistent, and the women questioned particularly the place and role of the GP therein: “GPs should explain better, I didn’t understand the seriousness of it, I thought it could be treated without any problem” (W21 USS); “My GP told me about it quickly, I didn’t see the point” (W11 USS). Information inadequacy was also discussed in the context of mitigative actions against medical desertification: “Retired doctors come to the health center, otherwise we don’t have a doctor. So screening is not at the forefront” (W10 VSS); “The doctors deal with the subject for which we came. In 10 min, it’s done and dusted, so prevention goes by the wayside” (FG 3). Furthermore, when women are asked about their relationship with screening procedures in France, 51/56 of them said that the reminder letter was not enough for them: “I put the reminder letter aside, telling myself I was going to do it, and then I forgot about it” (W20 VSS); “The letter doesn’t solve the problem of scheduling an appointment with the health professional” (FG2); “It’s just an incentive letter. Apart from telling us to consult our health professional, which we don’t have, and saying that screening prevents 90% of cancers, we’re told nothing about the substance of this screening, what it really is” (W2 USS).

Other factors identified as having less influence on the screening process did, however, illustrate important realities of life to consider, such as organization (11.8%), through the issue of travel and lack of time which was sometimes at the heart of difficulties in CC screening: “I don’t have a driver’s license. All travel here must be anticipated” (W22 VSS), as was time management: “I have a fast-paced life: young mother, young entrepreneur. I’m sorely short of time” (W21 USS); “Frankly, I don’t have the time. I’m an executive in a large company. I’ve been transferred three times. My professional life comes before prevention” (W19 VSS).

The participants also pointed to the more intimate and subjective factor of modesty as a factor that discourages them from undergoing CC screening (17.2%), notably through the intrusive aspect of the procedure: “When you go to the gynecologist, the position and the speculum are highly violent and invasive” (W15 USS); “Some women are stressed about going to a health professional to strip. It’s not a pleasant situation (...) we avoid it in terms of modesty” (FG 4). Beyond this dimension, the question of culture played a part in the vision and importance that could be attached to screening: “I’m Tahitian, and screening is not at all part of our mentality. We say to ourselves, if you’re going to get cancer, you’re going to get it” (W6 VSS); “I think of Muslim women, always accompanied by a man. Screening is impossible for them” (W15 USS).

#### 3.1.2. Additional Input from Focus-Groups

Also reflecting the factors identified above, the focus groups enabled the exploration of specific data and provided information on how to improve screening conditions and information access. The discussions resulted in the emergence of three main themes, i.e., communication by healthcare professionals, advertising campaigns, and access to information from an early age.

With regard to the first theme, the women highlighted what they considered to be a failure on the part of healthcare professionals to communicate certain information: “I’ve been seeing a gynecologist for years, but he’s never explained to me why you have a smear test, or how you could be contaminated (...) We are not informed about contamination. We are informed when we are contaminated or when we have cancer” (FG 4). The focus groups also revealed that this lack of communication could be linked to a lack of understanding. In detail, they described what they saw as a lack of accessibility to medical discourse, particularly in terms of their difficulty in understanding medical elements: “It’s obvious to them, with their medical discourse; we don’t know what they’re talking about” (FG 3).

A second emerging theme was the notion of the advertising campaign. This theme addressed the issue of inappropriate communication in a different way. According to the women, the information provided does not have to be the same for all age groups. In their view, the channel through which information is disseminated can influence women’s awareness of CC screening benefits: “videos with testimonials, billboards in towns and cities, via social networks for younger people” (FG 1); “We should do campaigns like those for AIDS in the 90s, short, punchy ones to hook young people. Or spots like those for road safety, which have a real impact. But don’t stick to the same image, because once you’re confronted with it, you become desensitized” (FG 2); “Maybe use social networks for younger people, via influencers, who can in turn influence older generations” (FG 3); “General information, commonplace everywhere. Not medical information that is not understood by everyone” (FG 4).

Finally, the third and last theme identified by the women concerned access to information from an early age and for the whole population, particularly on elements not commonly known to the general public: “Talking about the issue of infertility, maybe that can have an impact on young people” (FG 2) and on every person’s responsibility in this cancer: “Teenage boys and men should be made to take responsibility. It’s not just women in life. It’s always women” (FG 3); “At school, we talk about STDs, AIDS, etc. We could add HPV” (FG 1).

### 3.2. Self Screening for CC

#### 3.2.1. Levers for Self-Sampling

**Figure 2:**
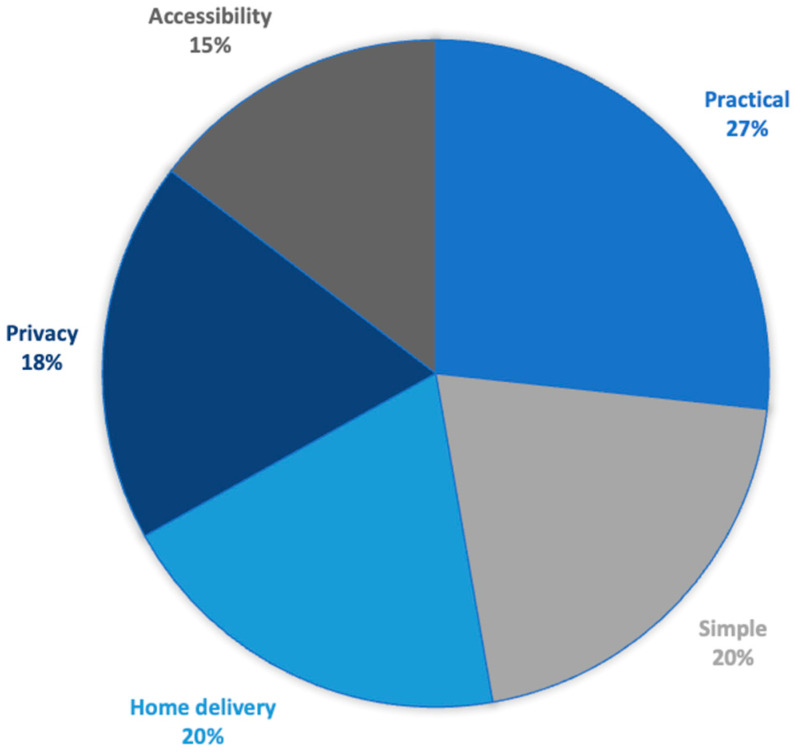
Levers that facilitate acceptance of self-sampling according to the interviewed women.

Figure 2 illustrates the five aspects identified by the participants, based on their experience with the home-delivered self-sampling kit, that are positives for self-testing. The importance of the themes raised by women is indicated by percentages.

The practical nature of the kit was most often mentioned by the participating women (27%): “It’s practical: we do it, we mail it, and we don’t talk about it anymore” (W3 VSS). In particular, this practical aspect effectively addressed the barriers of material organization and medical desertification: “There’s a practical side, no need to travel, no need to jump through hoops for an appointment” (W17 USS); “These are not the same tools as doctors, there’s no speculum, it’s better” (W13 VSS).

The participants pointed to the simplicity of the self-testing kit (20%) in terms of understanding and accessibility: “I found it clear and easy” (W21 VSS); “It’s much simpler than going to see a doctor” (W3 USS); “It was very clear, highly easy to use” (W1 USS).

The self-sampling kits also enabled a privately done act, testifying to the importance of respecting modesty (18%) and removing the barrier of sampling perceived as intrusive: “I’m in my own privacy with no one around” (W15 USS); “There’s less embarrassment in doing it yourself” (W24 USS); “Screening concerns our deepest intimacy, and the kit respects that” (W7 VSS). Sending the kit to women’s homes aligned with the notion of intimacy identified by the participants (20%). It also had a promotional effect and acted as an enabler of adherence to screening: “Sending it to the home will get a lot of women to adhere” (W5 VSS); “I did it because I received it at home” (W4 USS); “Multiple reminders for screening. I put it off, I put it off, then I received the kit at home” (W11 VSS).

This self-sampling kit also offered another real advantage in terms of accessibility (15%), whether in terms of culture or religion: “If you’re not at ease with your body, in terms of religion it removes barriers, you don’t have to ask permission. It removes all barriers” (W6 VSS); “It’s better for Muslim women in particular, they can use it away from the gaze of men, and often they can’t be examined because the doctor is a man, it’s a real plus” (W20 USS). Finally, doing the test oneself appeared to bring real personal satisfaction and enhance the woman’s self-esteem: “I felt more reassured about myself, doing it myself. It gave me more self-confidence than if a doctor had ordered me to do it” (W1 USS); “Doing it yourself is comforting” (W2 USS); “Narcissistically, it’s hyper-valorizing to be able to do it yourself” (FG 3).

#### 3.2.2. Barriers to Self-Sampling

Despite its many advantages, the participants did report limitations with self-sampling, underlining three factors in particular: possible difficulties in understanding the self-sampling kit, the results sent by mail, and reliability depending on the type of kit.

Firstly, the participants’ understanding of the kit and knowledge of their anatomy, both in theory and in practice, were evoked. This concerned both sociocultural aspects: “I think that sociocultural level plays a part in the use of the kit, in the question of hygiene, knowledge, the act, the interest of the thing, etc.” (W24 USS) and language aspects: “With the drawings, it helps some people a lot, especially when they don’t speak much French” (W7 VSS); “The picture speaks a lot more than the text” (W6 USS); “Pictures speak much louder than text” (FG2), “Maybe using a color code, for women who aren’t at ease with French, would be good. This color goes into that color. Take up the principle of pictograms” (W12 USS). This question of understanding how to use the self-testing kit called into question the relationship women have with their own bodies: “Many women need a professional, because I think that many women don’t know their bodies” (W9 USS); “Many women today don’t know the difference between their vagina and their anus. Ignorant women won’t know how to do it well” (FG 1).

The participants were also concerned about the mailing of results. For negative results, it was mentioned as being appropriate. However, for positive results, the women felt that not being accompanied by a health professional and finding themselves alone with this news was problematic: “Receiving only a piece of paper as a result is not acceptable” (W24 USS). Women particularly regretted a lack of clarity in the information provided with positive results: “Having a positive HPV test doesn’t mean you have cancer, but that’s not explained in the results letter” (FG 1); “With the results, you feel a bit alone. Improve the way results are sent out, with reassurance” (W18 VSS); “The results need to be written clearly, so that it’s understandable. So that we can understand. We’re not doctors” (W5 USS); “It’s not the fact of being confronted with a positive HPV test or the disease that scares us, it’s what this potential disease will do to our daily lives” (FG 4).

Finally, many of the women who had received a VSS kit questioned the reliability of the USS one: “I have more confidence in the vaginal test in terms of reliability” (W2 VSS); “I don’t see what can be detected in the urine sample. If I had received it, I wouldn’t have done it” (W10 VSS); “Urine is less reliable, it’s less detectable, there’s no material” (W9 VSS). Some of the women who performed the USS test were concerned about how it was transported: “I was worried about the transfer of the sample” (W20 USS); “I think transport can have an impact on the quality of the result” (W24 USS).

#### 3.2.3. Age Group Differences

Age, in particular, had a noticeable influence on women’s opinions concerning self-sampling.

Younger women (aged 30–40) were more likely to opt for USS if offered a choice between the two kit modalities (8 out of 11 women): “The urine kit looks like something you’d use to urinate at a festival, so it’s perhaps more suited to our generation” (FG 3); “Despite fears about sample transfer, we’re more used to doing urinalysis, it’s simpler, quicker, it’s in keeping with the rest” (W23 USS); “If the urine kit is as reliable, I’d choose the urine kit” (W21 USS).

Older women (aged 41–65) who received the USS kit were more afraid of making mistakes (28/45 women): “I was afraid of taking the sample incorrectly” (W7 USS), “I was afraid of taking it incorrectly, with all those utensils” (W12 USS). However, even if they had received the VSS kit, these same fears would have persisted: “So I would stay with the urine kit because, with the vaginal one, I would be even less sure of having taken the sample correctly” (W8 USS); “I would be afraid of doing the vaginal kit incorrectly, of not going far enough” (W15 USS).

In contrast, the older women who had received VSS kits showed less preference for one test over the other (36/45 women): “It doesn’t matter which test, as long as you get it at home” (W5 VSS); “The most important thing is the result, no matter if it’s vaginal or urinary” (W20 VSS).

#### 3.2.4. Adaptation of Kit Delivery to Women

The participants suggested that the delivery of VSS and USS kits could be adapted to suit the population. They pointed out that the VSS kit may still be perceived as invasive by some women: “I’m thinking of women who have been traumatized by having their samples taken at the gynecologist’s office; the vaginal may bring back fears of intrusion” (W5 VSS). Some also thought that it may be necessary in some cases to send the USS directly: “The urine kit is better suited to certain cultures, it’s less intrusive” (W18 VSS).

However, the home delivery itself of the kits did strongly raise understanding issues for some participants: “The kit should be given face-to-face with explanations. It would be more effective for some women” (W23 USS); “Some people need to see a professional, they can’t do it alone” (W16 VSS).

## 4. Discussion

The aim of the work we present here was to gather women’s opinions on CC screening and, in particular, on two experimental self-sampling strategies designed to reach under-screened populations in three rural French administrative departments with low medical density and/or low screening participation rates.

Numerous factors impeding CC screen have been identified, including lack of access to medical care, medical attitudes, and the ineffectiveness of provided information. Accessibility difficulties include both the scarcity of healthcare provision in priority intervention areas (PIAs) [26,40] and problems with territorial inequalities affecting screening opportunities [41]. The important provision-scarcity issue in PIAs calls into question the transmission of information by healthcare professionals, while these latter must imperatively be supported, particularly in rural areas [42]. Our study shows that in rural settings, few healthcare professionals propose screening to female patients, especially general practitioners, whereas their participation is critical for improving access to it [40].

Cultural aspects also stood out in our survey. These aspects are thought to have a major influence on the extent to which women undergo screening and the importance they attach to the procedure. Muslim culture was cited and rightly associated by our participants with a reduced likelihood of being up to date with cervical cancer screening [43]. Both interviews and focus groups validated the idea of accompanying certain immigrant women in screening education and offering appropriate support in follow-up to reduce disparities [44]. Level of education strongly influences the use of screening, with more highly educated women more likely to undergo it regularly [45]. Although social and socio-educational work pertinent to the local area is needed, prioritizing that aspect in public health policies may be questionable, when at least our participants clearly identified incomplete or even non-existent information transmission as a major issue (51/56 women). Moreover, even when information is provided, it is neither explicit nor systematically adapted (56/56 women). Information needs to be tailored to each woman and each situation. However, healthcare professionals, as reported by our participants in the interviews, seem reluctant to support the principle of proportionate universalism [46]. Readjusting the prevention policy could be a strategy for increasing screening. Our study shows that prevention needs to be accessible and comprehensible to all, with no break in the transmission of information. For example, according to our participants, conveying information exclusively during European Cervical Cancer Prevention Week does not leave a lasting impression. Our study also highlights the need to adapt prevention campaign information according to age and to the means by which it is disseminated (television, posters, social networks).

In addition to sociocultural and educational factors, modesty was another factor raised by the participants, who pointed to the difficulty of coping with a difficult and intrusive gynecological examination. The discomfort of the examination, particularly the use of the speculum, is regularly mentioned [47]. Our study appears to reinforce information suggesting that speculum-free sampling methods may represent a path toward increased CC screening [48].

Our participants validated CC self-screening via a home-sent kit, lending credence to several other studies suggesting that such an approach is more effective and cost-efficient among unscreened women than is relying solely on follow-up letters [14,49]. One of the most important benefits of self-sampling may be its ability to provide access to screening within certain cultures. Self-sampling has been shown to be widely accepted over several ethnic groups [50], and it should remove the obstacle of needing authorization from a husband or other male figure, noting, however, that some authors have reported husband opposition even to self-sampling in certain regions of the world [51].

As concerns limitations in self-testing, our participants underlined the need to simplify the kits as much as possible, and the USS kit in particular, to make it easier for people with language barriers to use them [52]. The reliability of USS was also widely questioned, despite its proven effectiveness in detecting HPV [20,21]. Reassuring women about the reliability of USS should be a priority if it is to be used in screening programs, particularly by explaining to them why the first-void urine can be used to collect HPV-containing mucus and debris from exfoliated cells from the female genital organs lining the urethra opening.

The participants in our study showed concern for poorly educated women who may have little knowledge about their anatomy and lower knowledge on CC and CC screening, and indeed, screening programs should address this potential problem [53]. Moreover, educational interventions have already been shown to increase CC screening [54]. Thus, in certain cases, a face-to-face meeting with a healthcare provider to explain the use of a self-sampling kit would be advantageous, and, furthermore, this tactic has already been shown to increase participation rates [14].

Beyond that potential need for assistance before taking the test, there was also a clear desire for more support when the results are received. Women with a positive HPV test received an explanatory letter with their results. This result was also sent to the attending physician if the declaration had been made to the health insurance. Several points were made in the explanatory letter: First, the fact that a positive HPV test may indicate a recent HPV infection or a persistent HPV infection that may already have caused lesions on the cervix. Secondly, it was strongly recommended to make an appointment with a general practitioner, a gynecologist, or a midwife to take a cervical test as soon as possible. Finally, it was announced that analysis of the cells from this cervical test would confirm whether or not cervical lesions were present, and that appropriate treatment would be offered. Despite this information, the issue most raised by participants was that of receiving their results by mail, and, above all, a lack of understanding.

Furthermore, it emerged from various interviews that women were perfectly aware of the implications of the self-screening process. They referred to a risk perceived less as a confrontation with illness or death but more as the danger of a “biographical rupture” [55]. For 44/56 women, there was a risk concerning what the outcome and potentially the illness could introduce in their daily lives: a break in habits, a break in projections, and so on. This potential biographical rupture led some women to develop mild but real manifestations of anxiety and identity disturbances, including questions about the meaning of their experiences, and unprecedented sensations of depersonalization comparable to those found in anxiety and borderline disorders. Furthermore, our study validates the potential of abnormal results to induce signs of post-traumatic stress [56]. Despite this anxious situation generated by the potential negative biographical turning points, various women nevertheless reported feelings of self-positivity linked to self-sampling (vs. conventional screening), i.e., a self-esteem-boosting sensation of having been active in the process, whatever the outcome. Nonetheless, for the majority, that sense of anxiety and identity fragility should be contained externally, i.e., by a reinforced professional presence to provide reassurance. All desired extra support when the results arrive.

As a result of our qualitative analysis, we present, in Box 1, our recommendations to improve participation in CC screening among women living in low medical density and/or low screening participation rates of France.

Box 1 **Recommendations to improve cervical cancer screening attendance in France as formulated by women in the CapU4 interviews.**

- To strengthen information by front-line professionals, especially GPs.
- To eliminate follow-up letters in priority intervention areas.
- To send self-sampling kits to women who have not responded to invitations.
- To deliver the kit to women’s homes address with explanatory leaflet (drawings to help you take the sample), rather than delivery to health professionals.
- To inform on the accuracy of testing on self-samples (vaginal or urinary kits).
- To explain in easy and understandable language how to use the self-sampling kit.
- To provide the choice for a urine kit to women who do not like using a vaginal kit (in particular, due to fear of intrusion, trauma, culture).
- Provide rapid medical support in the event of a positive HPV result.
- Develop information campaigns accessible to all (social networks, testimonials, striking images, etc.).

## 5. Conclusions

The European cancer plan is based on seven priority actions, including optimizing screening coverage to reach non-responders and empowering women in the process [57]. This qualitative study highlighted the need for this dual objective of optimization and empowerment in the context of CC screening to be underpinned by access to screening for all women, a better understanding of the issues among women, and the availability of health professionals within the process to alleviate women’s fears.

In PIAs, self-sampling is a pertinent option for hard-to-reach populations, in line with HAS guidelines. It would be appropriate to extend this strategy to all PIAs while adapting it to women’s profiles and ages and developing their autonomy by giving them the tools they need to carry it out. In fact, our study shows that the USS is preferred by younger women and would be better suited to women who are unfamiliar with their anatomy or whose religion is a barrier to screening. Finally, CC screening program in France is likely to remain inadequate in view of the limitations raised by this study. Therefore, it is imperative to carry out further projects on self-sampling strategies to promote women’s education and health professionals’ knowledge of cervical cancer, as well as to increase cervical cancer screening coverage.

## Figures and Tables

**Table 1:** Sociodemographic characteristics of study population.

	30–40	41–50	51–65	Rural	Urban	LTC
**VSS**	6	15	7	19	9	5
**USS**	5	14	9	23	5	8

VSS: vaginal self-sampling; USS: urinary self-sampling; LTC: long-term condition.

## Data Availability

The data presented in this study are available on request from the corresponding author. The data are not publicly available due to privacy and ethical restrictions: the validated ethics committee—2021-123, 25 November 2021, France—did not include making the data available to the public.

## Appendix A. Semi-Structured Interview Guide for Women

- Screening and you:
  (1) What made you decide to practice self-testing?
  (2) Did you know about this kit before being asked?
  (3) Were you able to talk to anyone close to you about it? Who were they?
  (4) Have you read about it (on the Internet)? Listened to a radio or TV program?
  (5) Have you talked to your doctor or another healthcare professional about this?
  (6) How do you usually relate to different types of screening?
- Cervical cancer and you:
  (1) Do you think you have enough information about cervical cancer? If not, who should give it to you?
  (2) Do you feel concerned by this cancer?
  (3) Do you think the fact that it is located in an intimate part of your body influences your relationship with this cancer?
  (4) Have health professionals suggested screening during a medical consultation?
  (5) Are you satisfied with your gynecological care?
  (6) Are you afraid of treatment (laser, conization, etc.)?
  (7) What was your experience of having a smear test taken in the office? (Was the test intrusive? Bad experience?) Who performed it?
  (8) For you, is it good or bad to know the doctor who takes the smear?
- During self-sampling:
  (1) What did you think of the information provided in the document? Was it sufficient? What was missing?
  (2) Were you afraid of doing the procedure incorrectly? Of hurting yourself?
  (3) Were you worried about hygiene?
  (4) Were you embarrassed to take the sample yourself? Or even felt any pain?
  (5) Does the fact that the sample can be taken at home play a role in your acceptance of the procedure?
  (6) Do you think that home sampling is a good thing for all women? If not, what could be done?
  (7) Do you think it’s as reliable as a smear test?
  (8) Is this kit sufficiently reliable in your opinion? Has there been sufficient proof of its reliability?
  (9) Is this type of self-examination in line with your culture? The culture of all the women you know?
  (10) Does this type of self-practiced examination suit you? Why or why not?
  (11) How did you feel when you took the test?
  (12) What could be improved?
  (13) Is a urine sample or vaginal swab the same for you in terms of experience?
  (14) How would you rate your satisfaction with self-testing from 0 to 5? Why or why not?
- Before and after self-testing:
  (1) Do you feel that anything has changed in your relationship with your health as a result of taking this self-test? Do you feel more involved in your health? More responsible?
  (2) Do you feel more at ease with your intimacy?
  (3) Do you feel your relationship with illness is less complicated?
  (4) Does taking part in screening change your relationship with healthcare professionals?
  (5) Does it make you want to take part in other screening programs?
  (6) What expectations do you have of healthcare professionals in the context of this type of screening, and have they been met?
  (7) Did the trust you have in your healthcare professional play a role in your decision to undergo screening?
  (8) Between 0 and 5, how would you rate the quality of the information provided by healthcare professionals in the context of this screening? Why or why not?
  (9) Do you need other sources of information? Which ones?
  (10) What could be improved: in terms of communication? Among healthcare professionals?
  (11) Have you thought about the results?
  (12) Have you thought about follow-up after the results?
  (13) What would be the ideal post-test kit for you?
  (14) In your opinion, who would be the ideal referral professional for the screening of this cancer?
  (15) How much confidence do you have in your screening professional (0 to 5)? Who is this professional: midwife, general practitioner, gynecologist? Why or why not?

## Appendix B. Focus-Group Interview Guide for Women

- Topic 1: Cervical cancer.
  1. If I say “cervical cancer,” what comes to mind?
    Follow-up/deepening:
    - How do you feel about it?
    - What do you know about this cancer?
  2. What do you know about cervical cancer screening?
    Follow-up/deepening:
    - Do you know whether this screening is financially covered or reimbursed?
    - Would full financial coverage be an incentive to carry out screening?
- Topic 2: The function and use of the self-testing kit.
  1. How did you feel when you received this kit?
    Follow-up/deepening:
    - And specifically the word cancer?
    - Did it concern you?
  2. What did you think of the instructions for use?
    Follow-up/deepening:
    - Did you encounter any difficulties?
    - What helped? What did you rely on? Could it be simplified (color codes, pictograms, positioning)?
  3. What did you think of receiving the kit at your home?
    Follow-up/deepening:
    - Do you have any other shipping/retrieval options in mind?
  4. Which self-test do you think is most appropriate (vaginal or urinary)?
    Follow-up/deepening:
    - In your opinion, which is the most reliable?
- Topic 3: Widespread use of self-screening.
  1. Do you think self-testing should be offered to all women?
    Follow-up/deepening:
    - Do you think self-testing is suitable for all ages?
    - For all geographical areas?
  2. Do you think all cultures/religions could benefit from using this kit?
- Topic 4: The role of healthcare professionals.
  1. In your opinion, can the self-testing system be completely separated from regular gynecological check-ups (carried out by a gynecologist, general practitioner (GP), or midwife)?
    Follow-up/deepening:
    - Would you like an explanation on how to use the kit from a healthcare pro-fessional? Why (reassuring? frightening?)
  2. Does certain behavior on the part of healthcare professionals discourage you from carrying out screening? Which ones?
    Follow-up/deepening:
    - Does your doctor talk to you about cervical cancer screening?
    - Do you feel accompanied by your doctor for CC screening? Can you de-scribe how you feel when the question of screening is raised by you or your GP? How does it make you feel?
    - Do you have any ideas for improving your dialogue with your doctor on this subject?
- Topic 5: Prospects and future of screening.
  1. Following the proposal of this self-testing kit, do you see more interest in getting tested?
    Follow-up/deepening:
    - Do you feel concerned by this cancer (more present, more real)?
  2. How can we make screening a priority in women’s lives?
    Follow-up/deepening:
    - Do you have any suggestions for improving the chances of women being screened?
